# Prognostic Significance of Gene Signature of Tertiary Lymphoid Structures in Patients With Lung Adenocarcinoma

**DOI:** 10.3389/fonc.2021.693234

**Published:** 2021-07-26

**Authors:** Hong Feng, Fujun Yang, Lihong Qiao, Kai Zhou, Junfei Wang, Jiao Zhang, Tian Tian, Ying Du, Hong Shangguan

**Affiliations:** ^1^Cancer Center, Shandong Provincial Hospital, Cheeloo College of Medicine, Shandong University, Jinan, China; ^2^Cancer Center, Shandong Provincial Hospital Affiliated to Shandong First Medical University, Jinan, China; ^3^Department of Oncology Weihai Municipal Hospital, Weihai, China; ^4^Department of Internal Medicine, The People’s Hospital of Pingyi County, Pingyi, China; ^5^Department of Respiratory and Critical Care Medicine, Qilu Hospital of Shandong University, Jinan, China; ^6^Department of Translational Medicine, Genecast Biotechnology Co., Ltd, Wuxi City, China

**Keywords:** lung adenocarcinoma, tertiary lymphoid structures, gene signature, tumor mutational burden, driver gene mutations

## Abstract

**Background:**

Lung adenocarcinoma (LUAD) is a highly mortal cancer. Tertiary lymphoid structures (TLS) are ectopic lymphoid organs with similar morphological and molecular characters to secondary lymphoid organ. The aim of this study is to investigate the prognostic effect of a gene signature associated with TLSs, including B-cell-specific genes.

**Methods:**

Clinical data of 515 LUAD patients in the TGCA cohort were used to examine the relationship of TLS signature with immune microenvironment, tumor mutational burden (TMB), and driver gene mutations. Patients were divided into the TLS signature high group and TLS signature low group, and comparative analysis of survival and its influencing factors between the two groups was performed. The resulting data were then validated in the GSE37745 cohort.

**Results:**

TLS signature high group had significantly better overall survival (OS) and progression-free interval (PFI) as well as significantly higher infiltration of immune cell subsets, cancer immune cycle (CIC) signature except for immunogram score2 (IGS2), and expression of major checkpoint genes than the TLS signature low group. Notably, while TLS signature was not markedly associated with TMB and mutation frequencies of driver genes, there were significant differences in overall survival of patients with given mutation status of *EGFR*, *KRAS*, *BRAF* and *TP53* genes between the TLS signature high and low groups.

**Conclusion:**

This study provided evidence that LUAD patients with high TLS signature had a favorable immune microenvironment and better prognosis, suggesting that TLS signature is an independent positive prognostic factor for LUAD patients.

## Introduction

Lung cancer (LC) is the leading cause of cancer-related deaths worldwide, with non–small cell lung cancer (NSCLC) being the most prevalent subtype (1). Lung adenocarcinoma (LUAD) is the most common subtype of NSCLC, accounting for more than 40% of lung cancer cases (1). Given the reported 5-year overall survival rate of less than 15% in LUAD, identification and application of new molecular biomarkers are of importance for predicting prognosis and metastasis in LUAD (2). In recent years, immune checkpoint blockade has provided a new approach for cancer therapy and contributed to extending survival of NSCLC patients. However, clinical trials showed that only a subset of patients experienced clinical benefit from the therapy. Therefore, it is necessary to identify more biomarkers for improving precision immunotherapy in NSCLC patients (3, 4).

Tertiary lymphoid structures (TLSs) are ectopic lymphoid organs that develop at sites of chronic inflammation and have been identified in several different cancers (5), including lung cancer. TLSs have very similar structure and development with lymph nodes (6). Mature TLSs are composed of T cell zones and B cell zones, which contain germinal centers (GCs) and are surrounded by a T-cell zone (7). High endothelial venules (HEV) and mature dendritic cells (DC) are also present within TLSs. Unlike primary and secondary lymphoid-like structures, the formation of TLSs is dependent on antigenic stimulation and represents an ongoing adaptive immune response (8). TLS is usually present in infiltrative tumor margins and stroma and is thought to be a site of lymphatic recruitment and immune activation, usually forming in the presence of enhanced inflammation (8). Tumor-associated TLSs are usually correlated with a good prognosis. Previous studies have reported that both follicular B-cells and DCs in TLS were correlated with long-term survival, thus potentially serving as new prognostic biomarkers for lung cancer patient (9, 10). At present, detection and quantification of TLS remain a challenge. In previous studies, Hematoxylin and eosin (H&E) staining and immunohistochemistry (IHC) with multiplex selected markers were conducted to detect the TLS, while this approach was inconvenient to quantify TLS. Recently, transcriptomic analyses were used to determine TLS-associated gene signatures. For example, a gene signature was identified based on differential gene expression between different cases of melanoma. This gene signature could reflect tumors with TLS in melanoma patients and predict clinical outcomes of melanoma patients treated with immune checkpoint blockade (5). The 9 genes used in the signature were high expressed in CD20+CD8+ cells in melanoma, which represented the B cell and T cell within the TLS. This TLS signature correlated strongly with B cell signatures and single B cell markers, and recent study reported the importance of B cell in good response to ICB therapy and favorable outcome (11). Therefore, this signature may have the potential to predict the prognosis of LUAD patients.

The aim of this study was to determine the prognostic significance of this TLS signature in LUAD patients by using high-dimensional datasets in The Cancer Genome Atlas (TCGA). Here, we compared the differential results of survival curve analysis between patients with high TLS signature and those with low TLS signature, while examining the relationship of TLS signature with immune microenvironment, tumor mutational burden (TMB), and driver gene mutations. Furthermore, the predictive effect of TLS signature on LUAD patients in the TCGA cohort was validated using the GSE37745 dataset.

## Materials and Methods

### Data Collection and Processing

The gene expression profiles, SNV data and clinical information of 515 LUAD patients were downloaded from the Cancer Genome Atlas (TCGA) database (https://gdc.cancer.gov/about-data/publications/panimmune) (11).

GSE37745 dataset (https://www.ncbi.nlm.nih.gov/geo/geo2r/?acc=GSE37745) was used as the validation cohort. The gene expression profiles and survival data of LUAD patients in this dataset were obtained from Gene Expression Omnibus database (GEO, http://www.ncbi.nlm.nih.gov/geo).

The proportions of 28 types of immune cells in the tumor microenvironment were determined by using single-sample gene set enrichment analysis (ssGSEA) (12).

According to a previous study, the steps of the cancer-immunity cycle were described by the following eight axes of the immunogram score (IGS): IGS1, T cell immunity; IGS2, tumor antigenicity; IGS3, priming and activation; IGS4, trafficking and infiltration; IGS5, recognition of tumor cells; IGS6, inhibitor cells; IGS7, checkpoint expression; and IGS8, inhibitory molecules (13). The gene sets IGS1, IGS3, IGS4, IGS5, IGS6, IGS7, and IGS8 were used as previously described (13).

### Statistical Analysis

Statistical analyses were conducted using R software (version 3.4.2.). Data were expressed as the median and interquartile range (IQR). Differences between two groups were analyzed by Wilcoxon rank sum test. The Kruskal-Wallis test was used to assess differences among multiple groups. Differences in Overall Survival (OS) and Progression-Free Interval (PFI) between two groups were evaluated by Kaplan–Meier survival curve and verified by the two-sided log-rank test. The prognostic capability of the resulting risk score was assessed by singular and multiple Cox regression analysis. A two-sided p value of <0.05 was considered statistically significant.

## Results

### A Correlation of TLS Signature With OS and PFI in LUAD Patients

**Figure 1:**
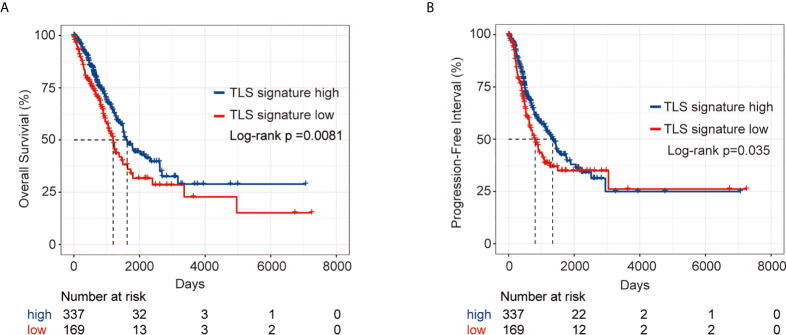
Survival analysis of LUAD patients in the TLS signature high and low groups from the TCGA cohort. **(A)** Kaplan–Meier plots of overall survival difference between tumors with TLS high (n = 337) and low groups (n = 169) in LUAD; **(B)** Kaplan–Meier plots of progression free survival difference between tumors with TLS signature high (n = 337) and low groups (n = 169) in LUAD.

We first comparatively analyzed the expression of nine genes in the TLS signature between LUAD tumor tissues and the corresponding normal tissues. As shown in **Figure S1**, there were significant differences in the expression of all nine genes except for CD1D between the two groups of tissues. Based on the expression pattern, LUAD patients were divided into a TLS signature high group (upper tertile) and a TLS signature low group (lower tertile). The differences in clinical characteristics between the two groups are shown in **Table S1**. We next compared the outcomes of patients between the TLS signature high and low groups. As depicted in **Figure 1**, the TLS signature low group had significantly poorer OS (P=0.0081) and PFI (P=0.035) than the TLS signature high group. We, therefore, conducted univariate and multivariate analyses using the Cox proportional hazard regression model to evaluate the impact of TLS signature and other clinicopathological factors on the survival of patients. As summarized in **Table 1**, univariate analysis identified pathological stage and TLS signature as significant predictors of OS (pathological stage: HR, 2.65; 95% CI, 1.95-3.62; P<0.001. TLS signature: HR, 0.79; 95% CI, 0.67-0.92; P=0.00231.) and PFI (pathological stage: HR, 1.623; 95% CI, 1.18-2.23; P=0.00284. TLS signature: HR, 0.84; 95% CI, 0.73-0.98; P=0.0268.). Likewise, highly expressed TLS signature was found to be a significant independent predictor of OS (HR, 0.72; 95% CI, 0.60-0.87; P=0.00049.) and PFI (HR, 0.74; 95% CI, 0.62-0.89; P=0.00104.) in the multivariate analysis.

**Table 1:** Univariate and multivariate cox analyses of prognosis of LUAD patients in the TCGA cohort.

	Univarate analysis		Multivariate analysis	
	HR (95% CI)	P value	HR (95% CI)	P value
OS				
Gender (male *vs* female)	0.94 (0.70-1.26)	0.672		
Age (>65 *vs* ≤65)	1.21 (0.90-1.62)	0.212		
Stage (III,IV *vs* I,II)	2.65 (1.95-3.62)	6.50E-10	2.36 (1.71-3.25)	1.89E-07
TLS_signature (high *vs* low)	0.79 (0.67-0.92)	0.00231	0.72 (0.60-0.87)	0.00049
Smoking_history (Yes *vs* No)	0.92 (0.61-1.39)	0.682		
PFI				
Gende r(male *vs* female)	0.93 (0.71-1.22)	0.605		
Age (>65 *vs* ≤65)	1.09 (0.82-1.43)	0.566		
Stage (III,IV *vs* I,II)	1.623 (1.18-2.23)	0.00284	1.44 (1.03-2.01)	0.03214
TLS_signature (high *vs* low)	0.84 ( 0.73-0.98)	0.0268	0.74 ( 0.62-0.89)	0.00104
Smoking_history (Yes *vs* No)	0.97 (0.66-1.45)	0.899		

### Relationship Between TLS Signature and Immune Microenvironment

**Figure 2:**
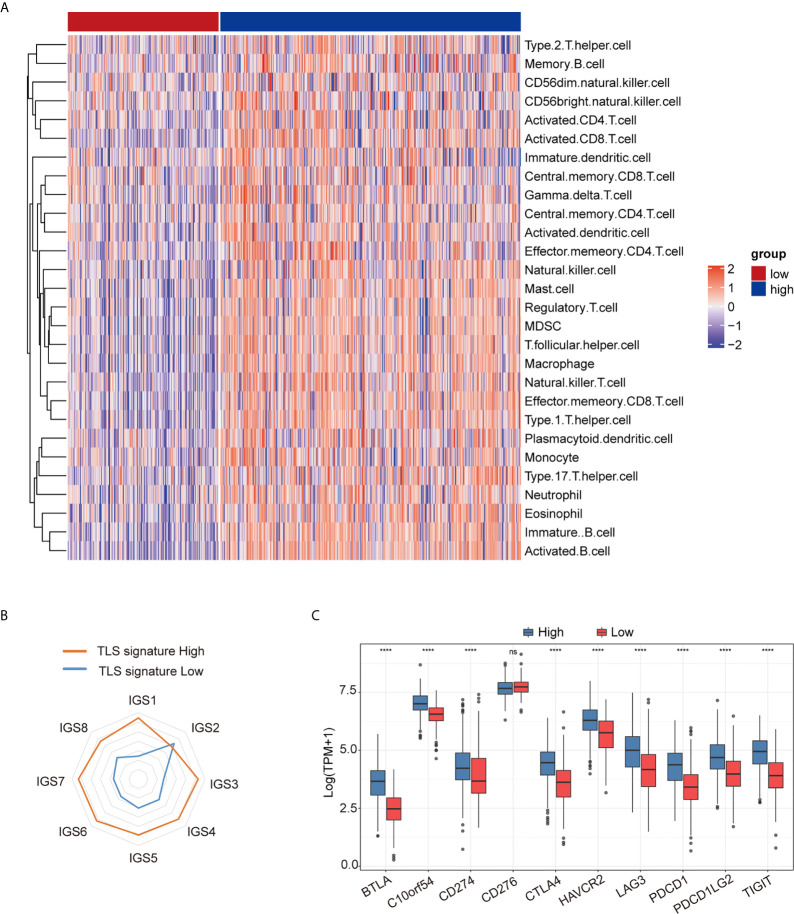
Relationship between TLS signature and tumor immune microenvironment. **(A)** Differences in immune cell subsets between the TLS signature high (n = 337) and low (n = 169) groups; **(B)** Differences in cancer immune circulation between the TLS signature high (n = 337) and low groups (n = 169); **(C)** Differences in the expression of checkpoint genes between the TLS signature high (n = 337) and low groups (n = 169). Wilcoxon text, ****P < 0.0001; ns, not significant.

As shown in **Figure 2A**, the degree of immune cell subset infiltration in the TLS signature high group was significantly higher than that in the TLS signature low group. We then analyzed relationships between the main immune cell subsets of TLS and TLS signature (**Figure S2**). As illustrated in **Figure 2B**, most of the cancer-immunity cycle (CIC) features except for IGS2 in the TLS signature high group were significantly higher than those in the low group. This observation was further supported by the heatmap (**Figure S3A**). The correlationships between TLS signature and individual features of the immune cycle are presented in **Figure S3B**. We further investigated the relationship between TLS signature and checkpoint gene expression. As shown in **Figure 2C**, the expression of major checkpoint genes, except for CD274, was significantly increased in the TLS signature high group compared with the low group. The relationships between TLS signature and checkpoint genes are illustrated in **Figure S4**.

### Relationship Between TLS Signature and TMB

**Figure 3:**
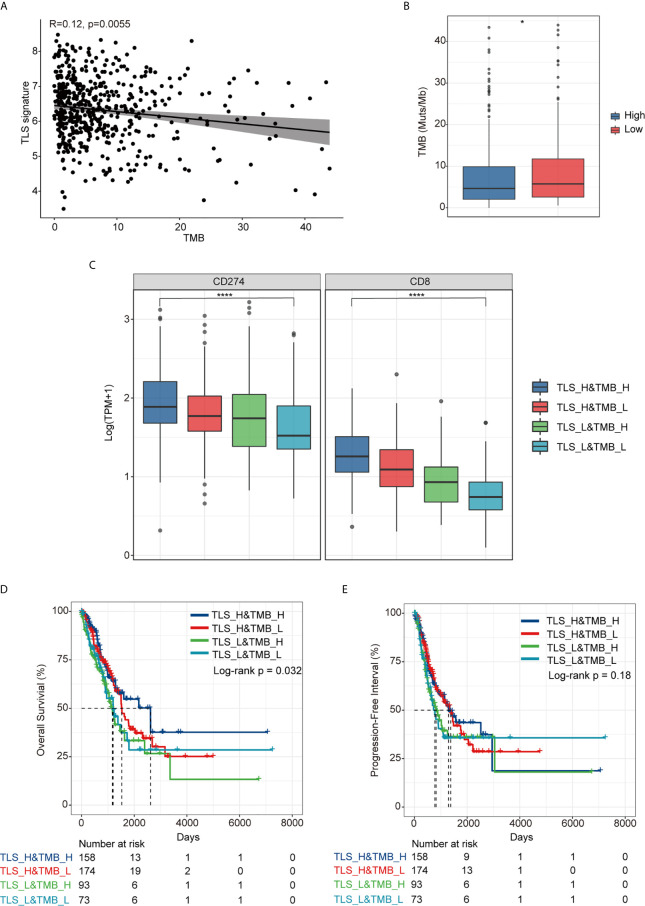
Relationship between TLS signature and TMB. **(A)** A correlation between TLS signature and TMB; **(B)** Differences in TMB between the TLS signature high (n = 337) and low groups (n = 169); **(C)** Differences in the expression of PD-L1 and CD8 gene among TLS high and TMB high group(TLS_H&TMB_H, n = 158), TLS high and TMB low group (TLS_H&TMB_L, n = 174), TLS low and TMB high group(TLS_L&TMB_H, n = 93), and TLS low and TMB low group (TLS_L&TMB_L, n = 73), Kruskal-Wallis test,*P < 0.05; ****P < 0.0001; **(D)** Differences in overall survival among the four indicated groups; **(E)** Differences in progression free survival among the four indicated groups.

As shown in **Figure 3A**, there was no significant correlation between TLS signature and TMB (r=0.12, p=0.0055), albeit the TMB in the TLS signature high group was slightly lower than that in the low group (p<0.05) (**Figure 3B**). For further comparative studies, we divided the patients into four groups (TLS_High&TMB_High, TLS_High&TMB_Low, TLS_Low&TMB_High, and TLS_Low&TMB_Low) based on the TMB level (with median value as cutoff) and the level of TLS signature. The comparative studies identified significant differences in the expression of CD274 and CD8 among all four groups (p < 0.0001) (**Figure 3C**). Moreover, we observed that there was a significant difference in OS among the four groups (p=0.032), with the best OS in TLS_H&TMB_H group (**Figure 3D**), whereas no significant difference in PFI was evident among the four groups (p=0.18) (**Figure 3E**).

### Relationship Between TLS Signature and Driver Gene Mutations

**Figure 4:**
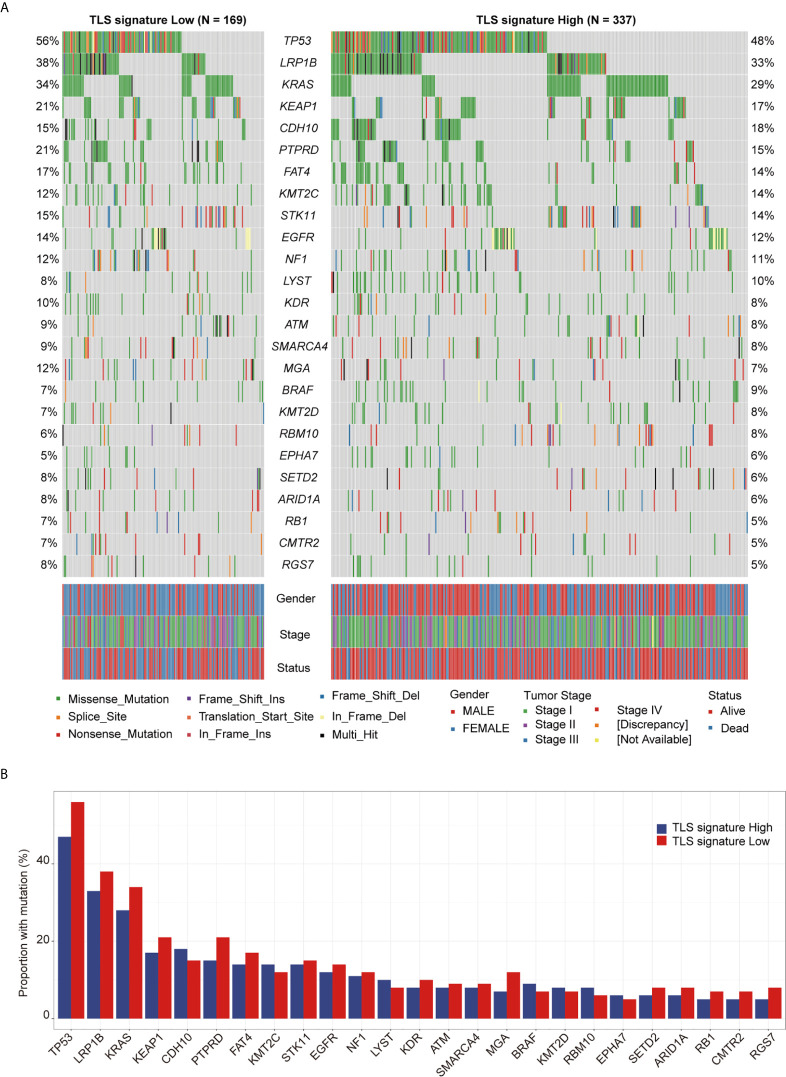
Relationship between TLS signature and mutation status of the driver genes. **(A)** Differences in the landscape of driver gene mutations between the TLS signature high (n = 336) and low groups (n = 169), the OncoPlot shows the significant mutated driver genes in LUAD tumors (≥5% in both groups); **(B)** Differences in mutational frequencies of top driver genes (≥5% in both groups) between the TLS signature high (n = 336) and low groups (n = 169).

**Figure 5:**
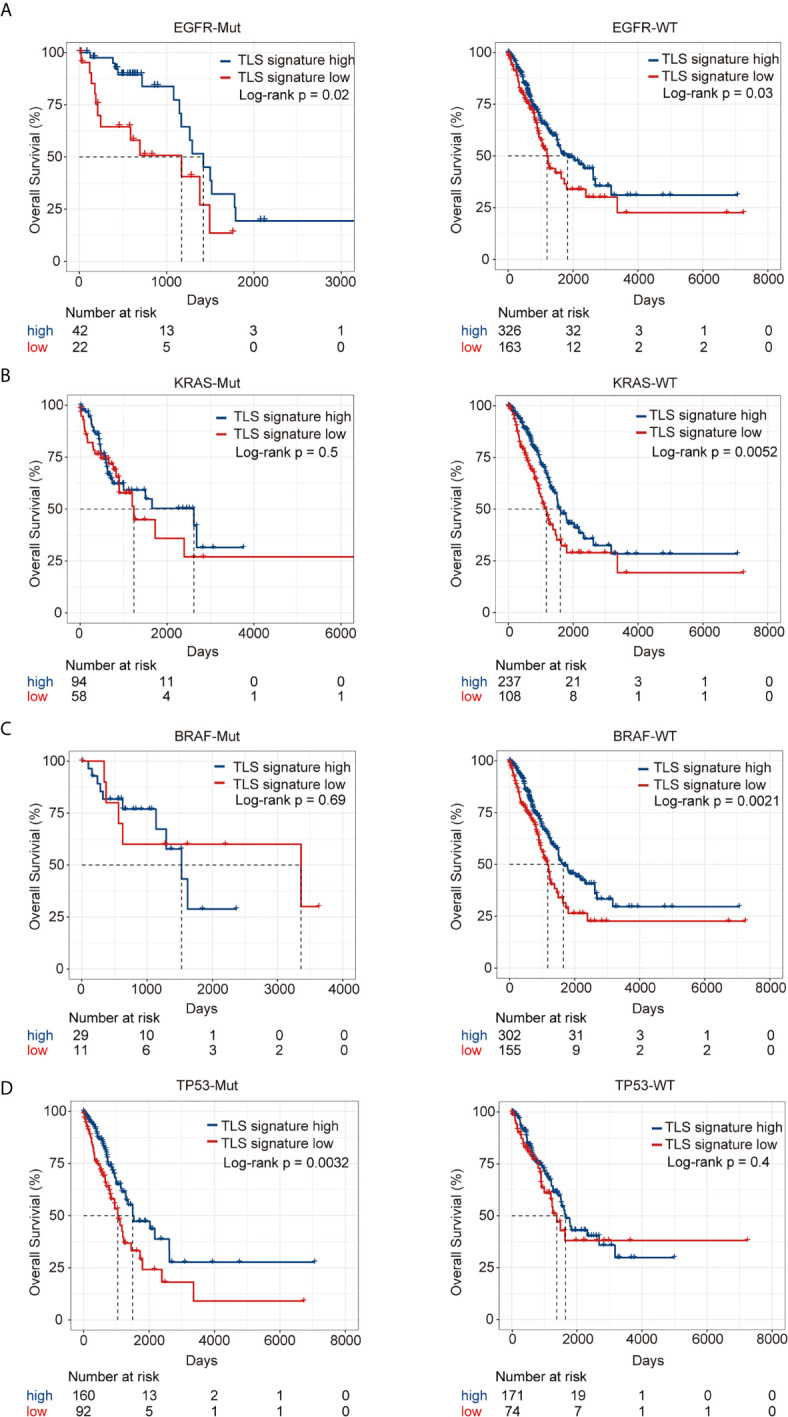
Relationship between TLS signature and OS of patients with given mutation status of driver genes. **(A)** Differences in OS of patients with wild type (high group n = 326, low group n = 163) or mutant EGFR (high group n = 42, low group n = 22) between the two indicated groups; **(B)** Differences in OS of patients with wild type (high group n = 237, low group n = 108) or mutant KRAS (high group n = 94, low group n = 58) between the two indicated groups; **(C)** Differences in OS of patients with wild type (high group n = 302, low group n = 155) or mutant BRAF (high group n = 29, low group n = 11) between the two indicated groups; **(D)** Differences in OS of patients with wild type (high group n = 171, low group n = 74) or mutant TP53 (high group n = 160, low group n = 92) between the two indicated groups.

The landscape of the driver genes with a mutation frequency greater than 5% in the TLS signature high group versus the TLS signature low group was illustrated in **Figure 4A**. Strikingly, there were no significant differences in the mutation frequency of each driver gene between the two TLS signature groups (**Figure 4B**). We next examined the relationships between the mutation status of the driver genes and OS or PFI of patients. As shown in **Figure 5**, while no significant differences in OS were found between the two TLS signature groups of patients with KRAS or BRAF mutations, OS of patients with wild type KRAS or BRAF in the TLS signature high group was significantly better than that in the low group (p=0.0052 and p=0.0021 for gene KRAS and BRAF, respectively). On the contrary, OS of patients with TP53 mutation in the TLS signature high group was significantly better than that in the low group (p = 0.0032), while there was no significant difference in OS between the two groups of patients with wild type TP53 gene. The relationships between mutation status of the driver genes and PFI of the two groups of patients are shown in **Figure S5**. Besides, patients with wild type or mutated EGFR in the TLS signature high group displayed a better OS than those in the low group. This finding was consistent with that observed in the cohort of LUAD patients. Notably, while there were no significant differences in OS and PFI between the two TLS signature groups of patients with EGFR-tyrosine kinase inhibitors (TKI)-sensitive mutation 19Del/L858R, patients without 19Del/L858R in the TLS signature high group had a better OS than those in the low group (**Figure S6**).

### Validation of the Predictive Effect of TLS Signature on LUAD Patients Using the GSE37745 Dataset

**Figure 6:**
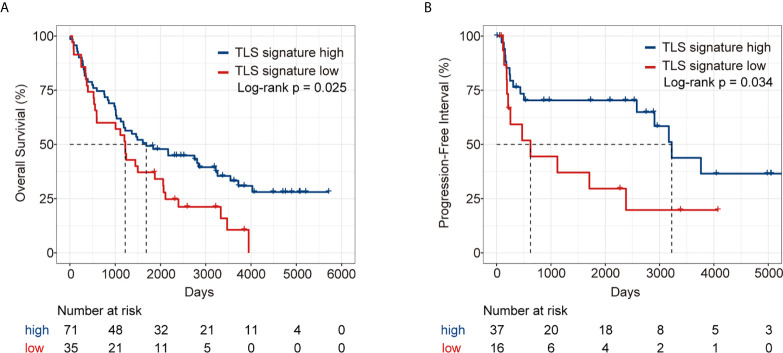
Survival analysis of LUAD patients in GSE37745 dataset between the TLS signature high and low groups. **(A)** Kaplan–Meier plots of overall survival difference between tumors with TLS high (n = 71) and low groups (n = 35) in LUAD; **(B)** Kaplan–Meier plots of progression free survival difference between tumors with TLS signature high (n = 37) and low groups (n = 16) in LUAD.

We further validated the predictive effect of TLS signature on LUAD patients in GSE37745 dataset. Patients in the dataset were divided into the TLS signature high group (upper tertile) and the TLS signature low group (lower tertile), and the comparative studies were performed on the two groups of patients. As illustrated in **Figure 6**, the TLS signature high group had significantly higher OS and PFI than the low group. Clearly, this observation was consistent with the finding in the TCGA cohort. The differences in basic clinical information between the two groups of patients are shown in **Table S2**. As expected, univariate analysis of this dataset identified TLS signature as a significant predictor of OS (HR, 0.59; 95% CI, 0.37-0.94; p=0.027) and PFI (HR, 0.44; 95% CI, 0.20-0.96; p=0.0388). Similarly, highly expressed TLS signature was found to be a significant independent predictor of PFI, based on the multivariate analysis of dataset GSE37745 (HR, 0.31; 95% CI, 0.11-0.83; P=0.0202) (**Table 2**).

**Table 2:** Univariate and multivariate cox analyses of prognosis of LUAD patients in the GSE37745 dataset.

	Univarate analysis		Multivariate analysis	
	HR (95% CI)	P value	HR (95% CI)	P value
OS				
Gender (male *vs* female)	0.79 (0.51-1.25)	0.316	0.27 (0.11-0.67)	0.0045
Age (>65 *vs* ≤65)	1.47 (0.94-2.30)	0.093		
Stage (III,IV *vs* I,II)	1.79 (1.01-3.17)	4.40E-02	6.18 (2.23-17.13)	4.60E-04
TLS_signature (high *vs* low)	0.59 (0.37-0.94)	0.027	0.46 (0.21-1.01)	0.053
Performance status (≥1 *vs* 0)	1.61 (1.02-2.53)	0.04	4.0 (1.72-9.32)	0.0013
Adjuvant treatment (yes *vs* no)	1.14 (0.58-2.27)	0.701		
PFI				
Gender (male *vs* female)	0.95 (0.42-2.08)	0.87	0.31 (0.09-1.08)	0.0654
Age (>65 *vs* ≤65)	0.95 (0.44-2.04)	0.89		
Stage (III,IV *vs* I,II)	1.72 (0.64-4.61)	0.28	2.99 (0.79-11.31)	0.1059
TLS_signature (high *vs* low)	0.44 (0.20-0.96)	0.0388	0.31 ( 0.11-0.83)	0.0202
Performance status (≥1 *vs* 0)	1.37 (0.62-3.00)	0.436	2.87 (0.99-8.31)	0.0524
Adjuvant treatment (yes *vs* no)	1.58 (0.71-3.52)	0.262		

## Discussion

TLS develops at sites of chronic infection or persistent inflammation in non-lymphoid tissues and has been observed in organ transplantation, autoimmune diseases and cancer (14). Multiple studies have shown that genes in TLS signature, such as CCR6 (15) and CD79B (16), can predict the prognosis of LUAD patients. Lin et al. identified TLSs as prognostic predictors in many cancers including lung cancer by analyzing genomic features associated with TLS formation in pan-cancer and their interactions with the tumor immune microenvironment (17). Consistently, the present study found that LUAD patients with high TLS signature displayed a better survival than those with low TLS signature, showing a marked association between TLS signature and the survival of LUAD patients. Moreover, we observed more favorable tumor immune microenvironment of patients in the TLS signature high group compared with the low group.

The prognostic value of the presence of TLSs have been reported for several times (9, 10, 19, 20). Most of them used IHC with one or two markers to evaluate TLS. IHC is not the golden standard of measurement of TLS, and there are several limits of this method. IHC could only detect a few markers to measure the presence of TLSs. However, RNA-seq could not only identify the presence of TLS and but also is ideal for high-throughput analysis. Therefore, analyzing a gene expression TLS signature *via* RNAseq was very useful to predict the prognosis of LUAD patients and perform high-throughput analysis at the same time. Another TLS-associated gene expression signature which have been reported was the 12-chemokine signature (21). But the 12-chemokine signature did not show significant prognostic value in lung cancers. The 12-chemokine signature included 12 chemokines, which were related to the neogenesis of TLSs, and the 9 gene TLS signature mainly represented the B cells and T cells within TLSs and strongly correlated with B cell markers. These two signatures represented different composition of TLSs and may represented with different types of TLSs. This may lead to the different predictive effects of different TLS signatures on survival. Therefore, the 9-gene signature, representing the TLS-associated gene expression signature, may show better prognostic value than the 12-chemokine signature in LUAD.

The tumor immune microenvironment is a complex network formed by different types of immune cell populations, which plays an important role in the development and progression of tumors while having a significant impact on prognosis or treatment outcome (17). Tumor development is influenced by the complex interactions between tumor cells and host immune responses within the tumor immune microenvironment (18). The tumor microenvironment harbors numerous immune cell subsets that are critically involved in anti-tumor immune response, and tumor-infiltrating lymphocytes are predictors of positive prognostic indicators and immune checkpoint blockade responses (19). Characterizing a TLS spatially, compositionally and functionally is an important step in describing the tumor immune microenvironment at a high resolution (20). In this study, we found that the immune cell subsets in the TLS signature high group were significantly higher than those in the TLS signature low group, while the levels of the major immune cell subsets in TLS were significantly positively correlated with TLS signature. Particularly, T cell immunity, priming and activation, trafficking and infiltration, recognition of tumor cells, inhibitor cells, checkpoint expression and inhibitory molecules were all positively correlated with TLS signature.

Interestingly, our data showed that the immunosuppressive subsets were also high in TLS signature high group, which may be correlated with poor outcome in some tumor types. TLS are very similar to lymph nodes in both structure and development, which includes many types of immune subsets (25). Immunosuppressive cells are also composition of TLS, therefore, high-level TLS may be indicative of enrichment of immunosuppressive cells. For example, Nikhil S et al. reported that in mice Treg cells accumulate in tumor-bearing lungs and located in within tumor-associated TLS (26). The associations of TLS with abundant immune subset have also been reported in melanoma, including immunosuppressive cells (27). In view of the above data, we inferred that the clinical outcome of LUAD patients may ultimately result from the delicate balance between pro- and anti-tumor immunity in the tumor microenvironment. The abundance of immunosuppressive cells may lead to the poor outcome of some patients in TLS-high subgroup. It should be noticed that, patients within the TLS-high group revealed increased expression levels of checkpoint molecules, and it is possible that these patients may also benefit from ICB immunotherapy. In addition, the biological roles and functional involvement of all immunosuppressive cells within TLSs remained to be clarified, and *in vitro* and *in vivo* functional studies will be helpful.

We also found increased expression of immune checkpoint gene in TLS signature high group. However high expression ICP was not always associated with poor outcomes. For example, Robert et al. reviewed several researches about PD-L1 expression in advanced NSCLC (28). In ten studies they reviewed, four reported a significant association between high PD-L1 expression and shorter survival (29, 30, 31, 32). Three studies found no significant association with survival (33, 34, 35), and one reported that patients with high PD-L1 expression experienced longer survival (36). There was also another study also showed that high-level TLS was associated with high expressed level and favorable clinical outcome (37). Although immune checkpoint inhibitors have been shown to have the significant clinical efficacy in human malignancies, most patients exhibit ab initio or adaptive resistance, indicative of the importance of identifying appropriate biomarkers and developing new therapies to overcome resistance (21). To date, a number of biomarkers have been identified as prognostic predictors for LUAD patients after receiving immunotherapy (22–24). And there was a study shown that the presence of TLS was associated with improved response to neoadjuvant anti-PD-1 in resected NSCLC (42). Here, we showed that the expression of major checkpoint genes except for CD274 in the TLS signature high group was significantly higher than that in the low group. According to the previous studies mentioned above, this finding suggests that TLS signature may have important implications in immunotherapy against LUAD.

Immune checkpoint blockade therapy targeting programmed cell death 1 (PD1) and PD1 ligand 1 (PDL1) has shown promising benefits in LUAD, and TMB is the most reliable biomarker associated with efficacy in the onset of PD-1-PD-L1 axis blockade in LUAD (25). High TMB has been reported to promote the accumulation of neoantigens on tumor cells and to enhance the activity of immune cells in the microenvironment, thus stimulating T-cell-dependent immune responses and inhibiting tumor development (26). While TMB could be another biomarker for effectively predicting prognosis of LUAD, its role remains under debate and is subject to further randomized trials for validation (27). The present study revealed that there were significant differences in overall survival among the four groups of LUAD patients classified based on both TLS signature and TMB, while patients with high TLS and high TMB had the best survival. Notably, no marked correlation between TLS signature and TMB was detected, albeit TMB in the TLS signature high group was slightly lower than that in the low group.

Gu et al. (28) investigated the relationship between TMB and gene mutations in LUAD patients of different ethnicities and found that Chinese have fewer gene mutations associated with high TMB than Caucasians, and LUAD patients with EGFR mutations have a better prognosis in Chinese population. The present study revealed no significant differences in the mutation status and mutation frequencies of the driver genes between the TLS signature high and low groups. On the contrary, there were significant differences in survival between the two TLS signature groups of patients with given mutation status of the driver genes. In these cases, OS of EGFR-TKI-sensitive mutation 19Del/L858R-negative patients in the TLS signature high group was significantly better than that in the TLS signature low group. EGFR/TKIs are the standard of care for LUAD patients with mutated EGFR, but the acquired drug resistance leads to inevitable disease relapse (29). Therefore, identifying a more suitable population for EGFR TKIs is important for the treatment of patients with EGFR mutant phenotypes. It has been found that B-cell infiltration is reduced in mutated KRAS enriched cluster, and overall survival is significantly decreased in LUAD patients with low B-cell infiltration compared with those with high B-cell infiltration (30). Notably, after receiving the combined administration of Nivolumab and Ipilimumab, advanced melanoma patients with wild type BERF display a lower survival rate than those with BARF mutation (31). It has also been shown that non-small cell lung cancer patients with TP53 mutations have a better OS after undergoing immune checkpoint blockade therapy (32). Consistently, the present study showed that patients without KRAS or BRAF mutations or patients with TP53 mutations in the TLS signature high group had significantly better OS than those in the TLS signature low group. Based on these observations, we propose that combined assessment of TLS signature and mutation status of the driver genes is useful for the selection of a population of LUAD patients for immunotherapy.

## Conclusion

This study identified a marked association between TLS signature and the survival of LUAD patients in both the TCGA cohort and GSE37745 dataset. Patients with high TLS signature had better survival than those with low TLS signature. While no difference in driver gene mutations was detected between the TLS signature high group and the low group, there was a significant difference in the survival of patients with given mutation status between the two groups.

## Data Availability Statement

The original contributions presented in the study are included in the article/**Supplementary Material**. Further inquiries can be directed to the corresponding authors.

## Author Contributions

HF and FY conceived and designed the study. LQ involved in data acquisition. KZ and JW analyzed and interpreted the data. JZ and TT involved in statistical analysis and drafted the manuscript. HS and YD revised the manuscript for important intellectual content. All authors contributed to the article and approved the submitted version.

## Funding

This article was supported by the Shandong Natural Science Foundation (NO. ZR2014HM063), Scientific and Technological Research project of Shandong Province (NO. 2011GGH21825) and Independent Innovation Plan of Universities in Jinan (NO. 201202047).

## Conflict of Interest

JZ and YD were employed by the Genecast Biotechnology Co., Ltd.

The remaining authors declare that the research was conducted in the absence of any commercial or financial relationships that could be construed as a potential conflict of interest.

## Publisher’s Note

All claims expressed in this article are solely those of the authors and do not necessarily represent those of their affiliated organizations, or those of the publisher, the editors and the reviewers. Any product that may be evaluated in this article, or claim that may be made by its manufacturer, is not guaranteed or endorsed by the publisher.

## Supplementary Material

Supplementary Figure 1 Comparative analysis of the expression of 9 TLS signature genes in tumor versus normal tissues in the TCGA cohort. Comparison of 9 gene expression of TLS signature between tumor tissue (n=515) and normal tissue (n=59). Wilcoxon text, *P < 0.05; **P < 0.01; ***P < 0.001; ****P < 0.0001; ns, not significant.

Supplementary Figure 2 Correlation of immune cell subsets with TLS signature. The Correlation between the TLS signature and 24 types of infiltrating immune cells was evaluated by Spearman Correlation analysis.

Supplementary Figure 3 Relationship between TLS signature and immune circulation. **(A)** Differences in CIC characteristics between the TLS signature high (n=336) and low groups (n=169); **(B)** Correlations between TLS signature and CIC characteristics.

Supplementary Figure 4 Heapmap of the expression of checkpoint genes between the TLS signature high and low groups. TLS signature high (n=336) and low groups (n=169).

Supplementary Figure 5 Relationship between TLS signature and PFI of patients with given mutation status of driver genes. **(A)** Differences in PFI of patients with wild type (high group n=289, low group n=144) or mutant EGFR (high group n=42, low group n=22) between the two indicated groups; **(B)** Differences in PFI of patients with wild type (high group n=237, low group n=108) or mutant KRAS (high group n=94, low group n=58) between the two indicated groups; **(C)** Differences in PFI of patients with wild type (high group n=302, low group n=155) or mutant BRAF (high group n=29, low group n=11) between the two indicated groups; **(D)** Differences in PFI of patients with wild type (high group n=171, low group n=74) or mutant TP53 (high group n=160, low group n=92) between the two indicated groups.

Supplementary Figure 6 Differences in OS and PFI between the TLS signature high and low groups with positive or negative EGFR-TKI-sensitive mutations. **(A)** Kaplan–Meier plots of overall survival difference between tumors with TLS high and low groups in LUAD with positive (high group n=29, low group n=14) or negative EGFR-TKI-sensitive mutations (high group n=13, low group n=8); **(B)** Kaplan–Meier plots of progression free survival difference between tumors with TLS high and low groups in LUAD with positive (high group n=29, low group n=14) or negative EGFR-TKI-sensitive mutations (high group n=13, low group n=8).

Supplementary Table 1 Basic clinical information of LUAD patients in the TCGA cohort.

Supplementary Table 2 Basic clinical information of LUAD patients in the GSE37745 dataset.
