# Supplementary figures and images for: Prognostic Significance of Gene Signature of Tertiary Lymphoid Structures in Patients With Lung Adenocarcinoma
Source: Front Oncol. 2021 Jul 26;11:693234. doi: 10.3389/fonc.2021.693234 (PMC8352557; doi:10.3389/fonc.2021.693234)

Figure S1

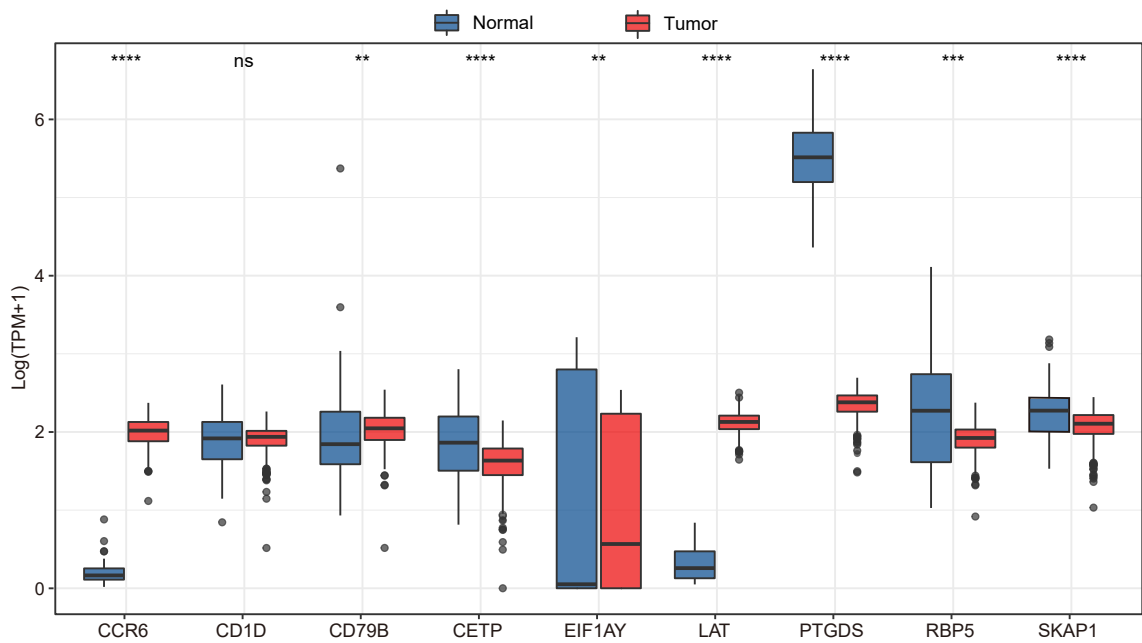

Supplement: Supplementary Figure 1 — Comparative analysis of the expression of 9 TLS signature genes in tumor versus normal tissues in the TCGA cohort. Comparison of 9 gene expression of TLS signature between tumor tissue (n=515) and normal tissue (n=59). Wilcoxon text, *P < 0.05; **P < 0.01; ***P < 0.001; ****P < 0.0001; ns, not significant. [file DataSheet_1.pdf]

Figure S2

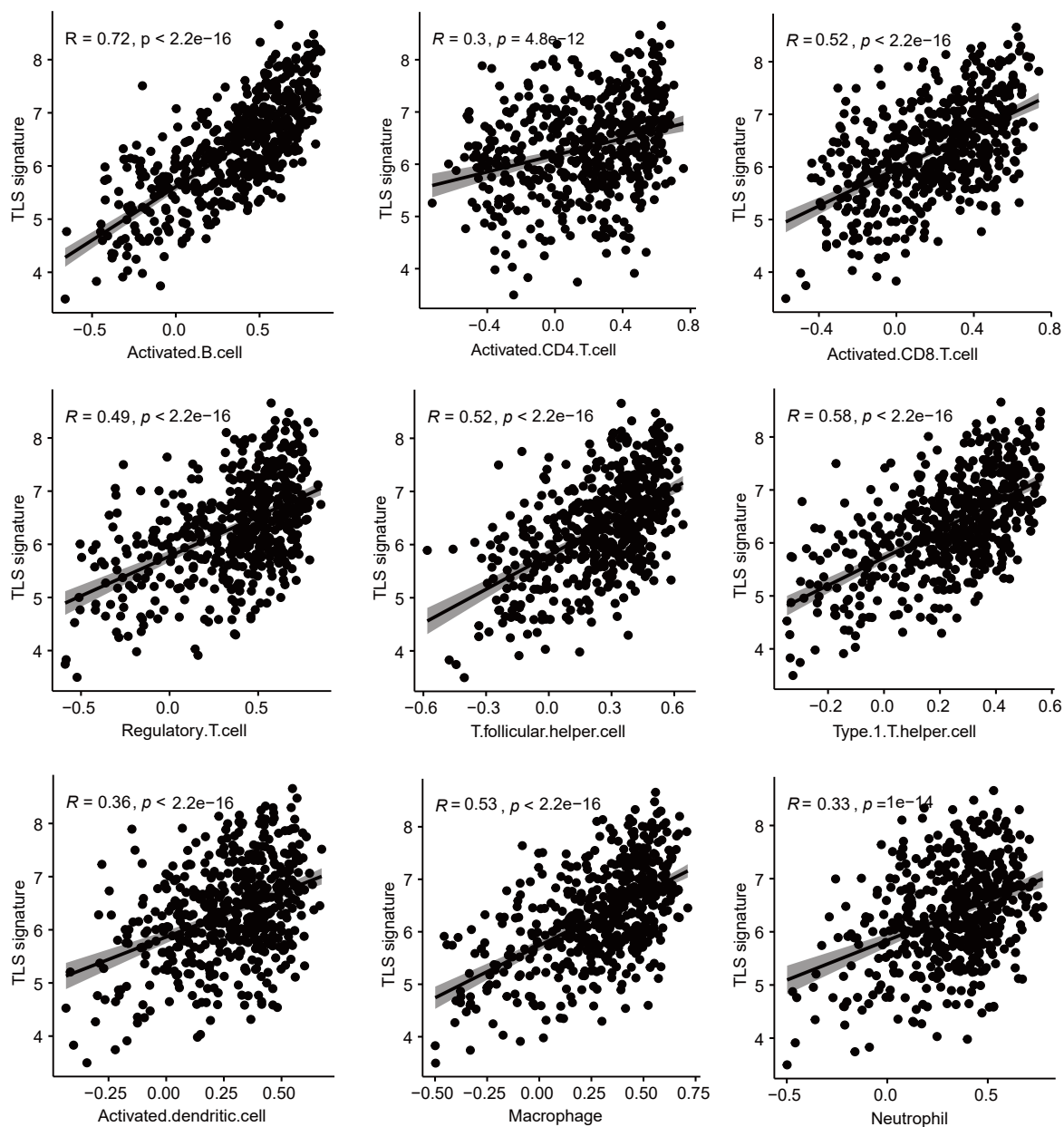

Supplement: Supplementary Figure 2 — Correlation of immune cell subsets with TLS signature. The Correlation between the TLS signature and 24 types of infiltrating immune cells was evaluated by Spearman Correlation analysis. [file DataSheet_2.pdf]

Figure S3

A

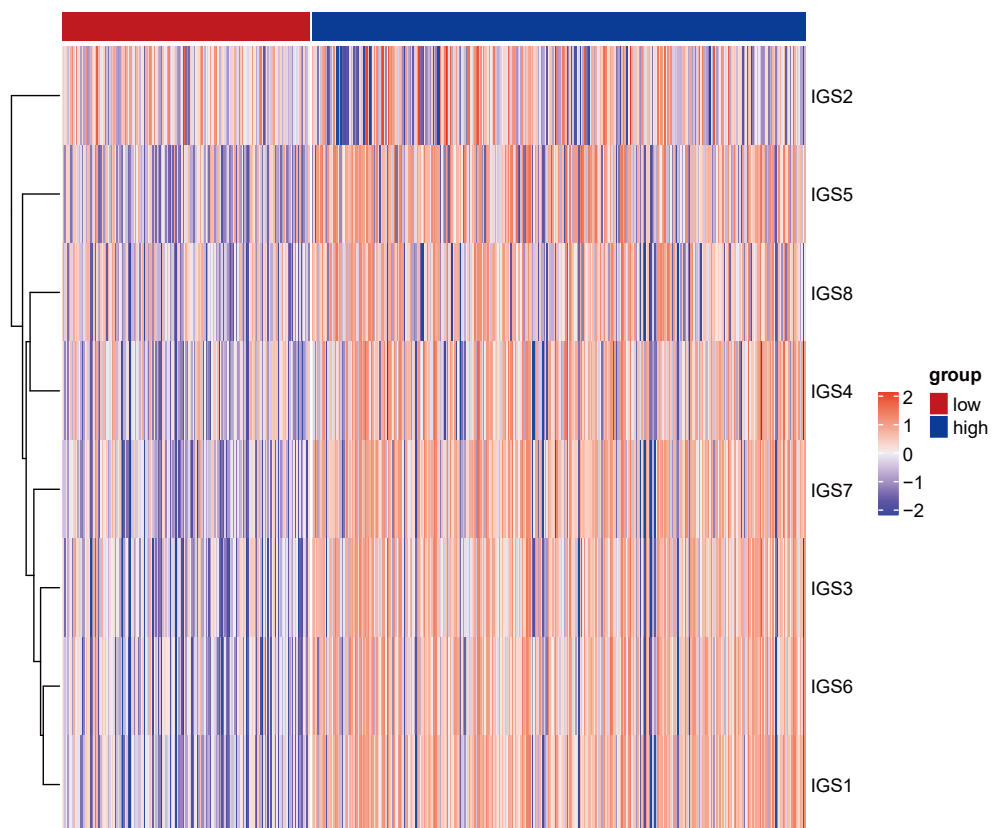

B

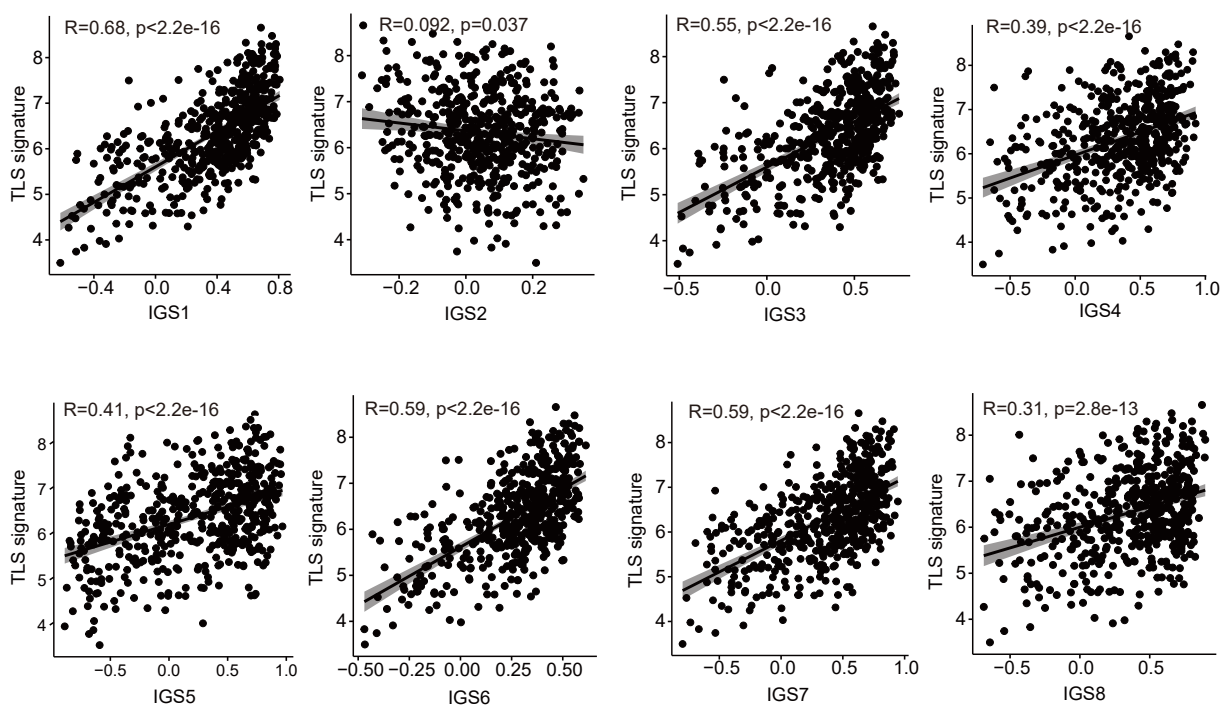

Supplement: Supplementary Figure 3 — Relationship between TLS signature and immune circulation. (A) Differences in CIC characteristics between the TLS signature high (n=336) and low groups (n=169); (B) Correlations between TLS signature and CIC characteristics. [file DataSheet_3.pdf]

Figure S5

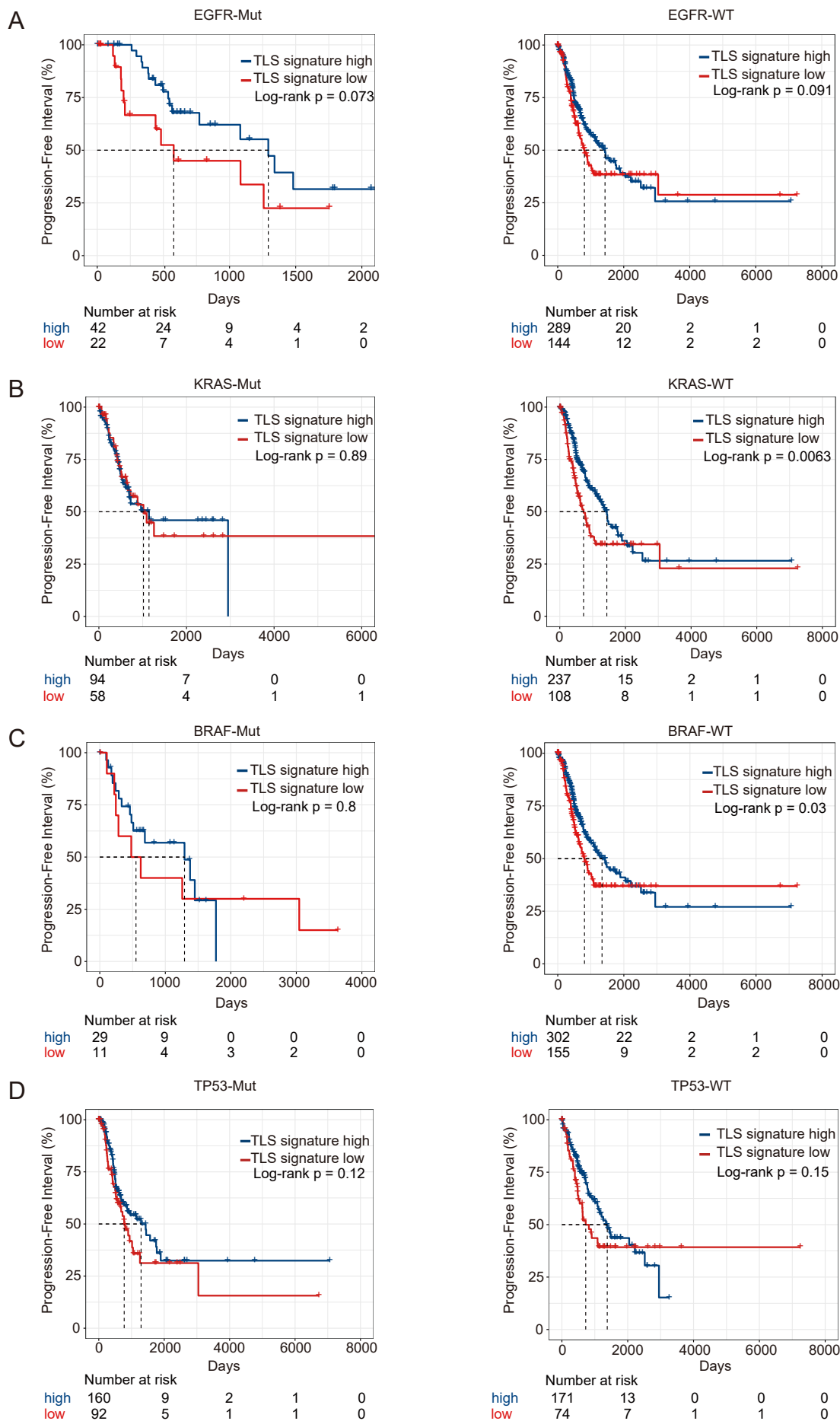

Supplement: Supplementary Figure 5 — Relationship between TLS signature and PFI of patients with given mutation status of driver genes. (A) Differences in PFI of patients with wild type (high group n=289, low group n=144) or mutant EGFR (high group n=42, low group n=22) between the two indicated groups; (B) Differences in PFI of patients with wild type (high group n=237, low group n=108) or mutant KRAS (high group n=94, low group n=58) between the two indicated groups; (C) Differences in PFI of patients with wild type (high group n=302, low group n=155) or mutant BRAF (high group n=29, low group n=11) between the two indicated groups; (D) Differences in PFI of patients with wild type (high group n=171, low group n=74) or mutant TP53 (high group n=160, low group n=92) between the two indicated groups. [file DataSheet_7.pdf]

Figure S6

A

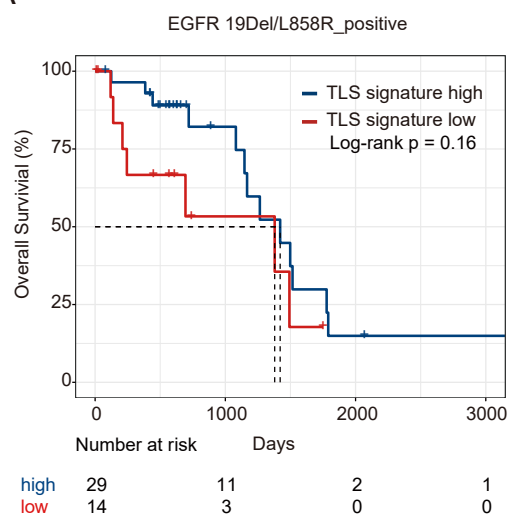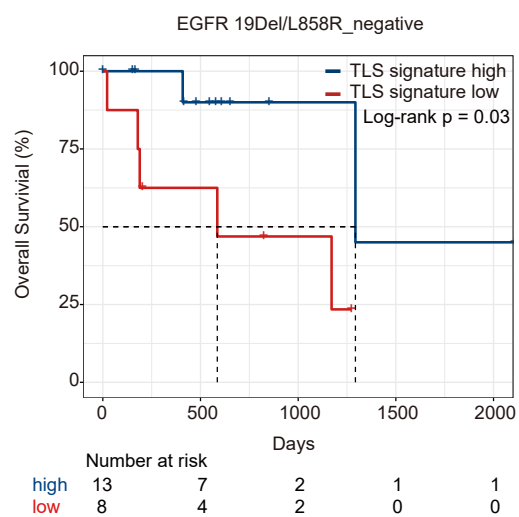

B

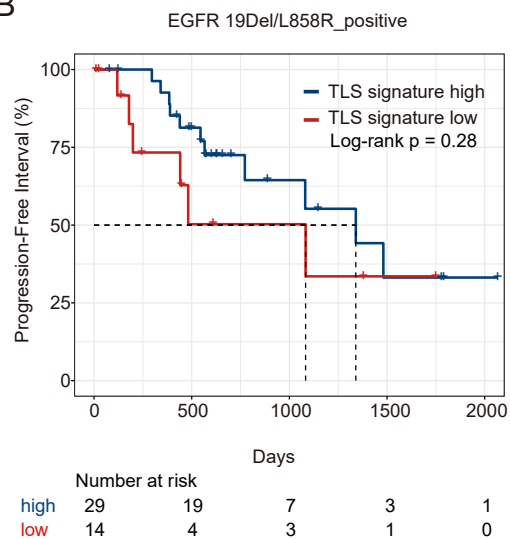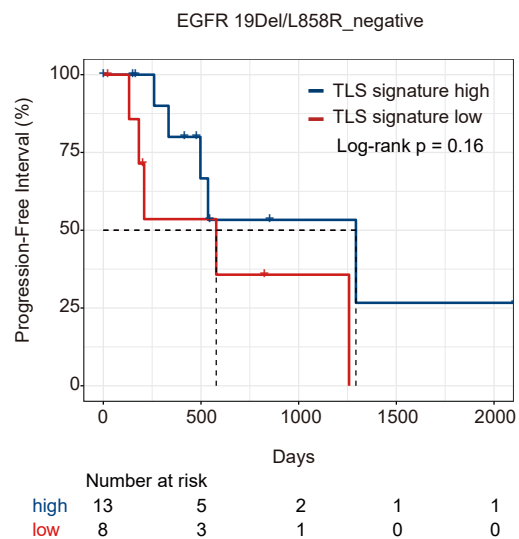

Supplement: Supplementary Figure 6 — Differences in OS and PFI between the TLS signature high and low groups with positive or negative EGFR-TKI-sensitive mutations. (A) Kaplan–Meier plots of overall survival difference between tumors with TLS high and low groups in LUAD with positive (high group n=29, low group n=14) or negative EGFR-TKI-sensitive mutations (high group n=13, low group n=8); (B) Kaplan–Meier plots of progression free survival difference between tumors with TLS high and low groups in LUAD with positive (high group n=29, low group n=14) or negative EGFR-TKI-sensitive mutations (high group n=13, low group n=8). [file DataSheet_8.pdf]
