# Supplementary material for: Prognostic Significance of Gene Signature of Tertiary Lymphoid Structures in Patients With Lung Adenocarcinoma
Source: Front Oncol. 2021 Jul 26;11:693234. doi: 10.3389/fonc.2021.693234 (PMC8352557; doi:10.3389/fonc.2021.693234)
Supplement: Supplementary Figure 4 — Heapmap of the expression of checkpoint genes between the TLS signature high and low groups. TLS signature high (n=336) and low groups (n=169). [file DataSheet_4.pdf]

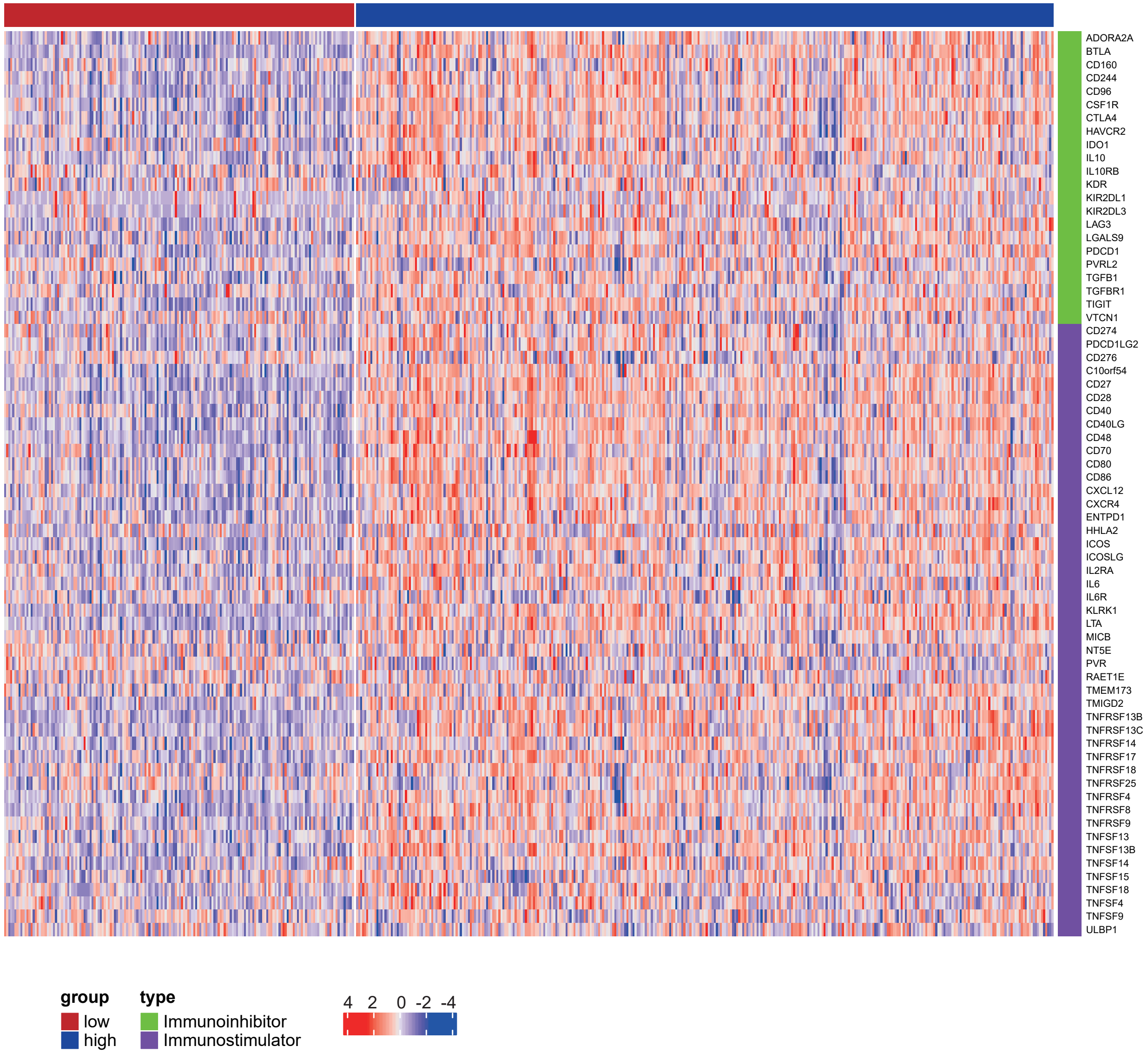
ADORA2A
BTLA
CD160
CD244
CD96
CSF1R
CTLA4
HAVCR2
IDO1
IL10
IL10RB
KDR
KIR2DL1
KIR2DL3
LAG3
LGALS9
PDCD1
PVRL2
TGFB1
TGFBR1
TIGIT
VTCN1
CD274
PDCD1LG2
CD276
C10orf54
CD27
CD28
CD40
CD40LG
CD48
CD70
CD80
CD86
CXCL12
CXCR4
ENTPD1
HHLA2
ICOS
ICOSLG
IL2RA
IL6
IL6R
KLRK1
LTA
MICB
NT5E
PVR
RAET1E
TMEM173
TMIGD2
TNFRSF13B
TNFRSF13C
TNFRSF14
TNFRSF17
TNFRSF18
TNFRSF25
TNFRSF4
TNFRSF8
TNFRSF9
TNFSF13
TNFSF13B
TNFSF14
TNFSF15
TNFSF18
TNFSF4
TNFSF9
ULBP1
group
low
high
type
Immunoinhibitor
Immunostimulator
4 2 0 -2 -4
