# Supplementary material for: Prognostic Significance of Gene Signature of Tertiary Lymphoid Structures in Patients With Lung Adenocarcinoma
Source: Front Oncol. 2021 Jul 26;11:693234. doi: 10.3389/fonc.2021.693234 (PMC8352557; doi:10.3389/fonc.2021.693234)
Supplement: Supplementary Table 1 — Basic clinical information of LUAD patients in the TCGA cohort. [file DataSheet_5.pdf]

Table S1

		TLS signature high (n=343)	TLS signature low (n=172)	P value
Age (mean (SD))		66.03 (9.55)	64.05 (10.56)	0.059
Gender (%)				
	Male	190 (55.4)	48 (27.9)	<0.001
	Female	153 (44.6)	124 (72.1)	
Tumor Stage (%)				
	I	195 (56.9)	80 (46.5)	0.032
	II	78 (22.7)	44 (25.6)	
	III	51 (14.9)	33 (19.2)	
	IV	12 (3.5)	14 (8.1)	
	Not known	7 (2.0)	1 (0.6)	
Smoking History (%)				0.533
	Yes	286 (83.4)	140 (84.9)	
	No	47 (12.2)	28 (13.4)	
	Not known	10 (2.9)	4 (2.3)	
Mutation Status				
EGFR (%)				
	Wild Type	177 (51.6)	74 (43.0)	0.824
	Mutation	160 (46.6)	95 (55.2)	
	Not known	6 (1.7)	3 (1.7)	
TP53 (%)				
	Wild Type	177 (51.6)	74 (43.0)	0.079
	Mutation	160 (46.6)	95 (55.2)	
	Not known	6 (1.7)	3 (1.7)	
KRAS (%)				
	Wild Type	241 (70.3)	111 (64.5)	0.214
	Mutation	96 (28.0)	58 (33.7)	
	Not Report	6 (1.7)	3 (1.7)	
BRAF (%)				
	Wild Type	307 (89.5)	158 (91.9)	0.449
	Mutation	30 (8.7)	11 (6.4)	
	Not known	6 (1.7)	3 (1.7)	
