# Supplementary material for: Prognostic Significance of Gene Signature of Tertiary Lymphoid Structures in Patients With Lung Adenocarcinoma
Source: Front Oncol. 2021 Jul 26;11:693234. doi: 10.3389/fonc.2021.693234 (PMC8352557; doi:10.3389/fonc.2021.693234)
Supplement: Supplementary Table 2 — Basic clinical information of LUAD patients in the GSE37745 dataset. [file DataSheet_6.pdf]

Table S2

		TLS signature high (n=71)	TLS signature low (n=35)	P value
Age (mean (SD))		63.62 (9.18)	61.57 (9.29)	0.282
Gender (%)				
	Male	41 (57.7)	5 (14.3)	<0.001
	Female	30 (42.3)	30 (85.7)	
Performance Status (%)				
	0	41 (57.7)	24 (68.6)	0.69
	1	24 (33.8)	9 (25.7)	
	2	4 (5.6)	2 (5.7)	
	3	2 (2.8)	0 (0.0)	
Tumor Stage (%)				
	I	50 (70.4)	20 (57.1)	0.238
	II	12 (16.9)	7 (20.0)	
	III	8 (11.3)	5 (14.3)	
	IV	1 (1.4)	3 (8.6)	
Adjuvant Treatment (%)				
	Yes	9 (12.7)	6 (17.1)	0.326
	No	30 (42.3)	10 (28.6)	
	Not known	32 (45.1)	19 (54.3)	
